# Approaches in type 1 diabetes research: A status report

**DOI:** 10.4103/0973-3930.53126

**Published:** 2009

**Authors:** Oindrila Raha, Subhankar Chowdhury, Samir Dasgupta, P. Raychaudhuri, B. N. Sarkar, P. Veer Raju, V. R. Rao

**Affiliations:** Anthropological Survey of India, 27-Jawaharlal Nehru Road, Kolkata, West-Bengal - 700 016, India; 1 Endocrinology Department, SSKM Hospital, Kolkata, India; 2 Rabindranath Research Institute of Cardiac Sciences, Kolkata, India; 3 Endocrinology Department, Calcutta Medical College and Hospital, Kolkata, India; 4 Human Genetics Department, Andhra University, Visakhapatnam, India

**Keywords:** Diabetes, HLA, Insulin

## Abstract

Type 1 diabetes is a multifactorial disease with an early age of onset, in which the insulin producing β cell of the pancreas are destroyed because of autoimmunity. It is the second most common chronic disease in children and account for 5% to 10% of all diagnosed cases of diabetes. India is having an incidence of 10.6 cases/year/100,000, and recent studies indicate that the prevalence of type 1 diabetes in India is increasing. However in view of poor health care network, there is no monitoring system in the country. Of the 18 genomic intervals implicated for the risk to develop type 1 diabetes, the major histocompatibility complex (MHC) region on chromosome 6p21.31 has been the major contributor estimated to account for 40-50%, followed by 10% frequency of INS-VNTR at 5’ flanking region of the insulin gene on chromosome 11p15.5. However, population studies suggest that > 95% of type 1 diabetes have HLA-DR3 or DR4, or both, and in family studies, sibling pairs affected with type 1 diabetes have a non-random distribution of shared HLA haplotypes. As predisposing genetic factors such as HLA alleles are known, immunological interventions to prevent type 1 diabetes are of great interest. In the present study we have reviewed the status of molecular genetics of the disease and the approaches that need to be adopted in terms of developing patient and suitable control cohorts in the country.

## Introduction

Diabetes mellitus is a metabolic disease, characterized by high glucose level in blood (hyperglycemia). Diabetes mellitus is divided into type 1 diabetes mellitus and type 2 diabetes mellitus. Type 1 diabetes, previously encompassed by the terms insulin-dependent diabetes mellitus (IDDM) or juvenile-onset diabetes, results from a cellular-mediated autoimmune destruction of the β cell of the pancreas.[1] The terms type 1 and type 2 –diabetes mellitus are retained, with Arabic numerals being used rather than Roman numerals.[2] The class, or form, named type 1 diabetes encompasses the vast majority of cases that are primarily due to pancreatic islet β cell destruction and that are prone to ketoacidosis. This form also includes those cases currently ascribable to an autoimmune process and those for which an etiology is unknown. It does not include those forms of β cell destruction or failure for which non-autoimmune-specific causes can be assigned (e.g., cystic fibrosis). In the majority of cases, type 1diabetes is the result of autoimmunity directed at the pancreatic β cells, known as *type 1A-Diabetes.*[3] A proportion of cases, however, does not have evidence of autoimmunity and are currently classified, according to the American Diabetes Association (ADA), as idiopathic, *type 1B-Diabetes*[2]. A minority of Asian or African type1-B patients suffers episodic ketoacidosis and has varying degrees of insulin deficiency between these episodes. This form of diabetes is strongly inherited, but lacks immunological evidence for β cell autoimmunity and it is not human leukocyte antigen associated.[4–6]

Traditionally type 1 diabetes was characterized by the presence of certain clinical presentation in blood and the age of onset. ADA 1997 has prescribed new guidelines based on pathogenic process and use of insulin requirement for the classification of type 1 diabetes. Type 1 diabetes is characterized by the presence of antibodies to a 65 kD Glutamic Acid Decarboxylase antigen (GAD), Insulinoma-associated protein-2 antibodies (IA-2 orICA512), which is now known as protein- tyrosine phosphatase (PTP),[7] insulin autoantibodies (IAAs), and islet cell autoantibodies (ICAs), in blood that identify the autoimmune process that leads to β cell destruction.[2]

Studies on human and animal models indicate MHC class II–mediated effects on the T cell repertoire in the thymus on disease susceptibility.[8] In humans, there are two major classes of T lymphocytes: CD8^+^ cytotoxic lymphocytes and CD4^+^ helper lymphocytes. CD8^+^ cytotoxic lymphocytes recognizes processed antigens bound to MHC class I molecules on the surface of cells (*e.g.* β cell), while CD4^+^ helper lymphocytes recognizes processed antigens bound to MHC class II molecules on the surface of antigen-presenting macrophages (APC)and dendritic cells. In type 1 diabetes, the direct cell-to-cell interaction between antigen-specific CD8^+^ cytotoxic T lymphocytes and autoantigens on β cells results in β cell killing. Antigen-specific CD4^+^ helper T lymphocytes act by recognizing autoantigens that have been picked up and processed by APCs expressing class II molecules. This indirect mechanism results in the release of a variety of effector molecules and is called as *bystander killing*. These events in the thymus could affect both the CD4 and CD8 T cell repertoires. Susceptibility may arise through positive selection of autoreactive T cells by positively associated class II alleles, whereas protection may occur via negative selection of T regulatory cells, mediated by negatively associated alleles.[9]

The *INS*-VNTR locus affects expression of the insulin gene and its precursors in the thymus. The destructive process leads to severe insulin depletion, which results in hyperglycemia, also an increase in fat breakdown and fatty acid oxidation, resulting in the excessive production of ketones. Some patients, particularly children and adolescents, may present with ketoacidosis at the first manifestation of the disease. If left untreated, these metabolic disturbances lead progressively to central nervous system depression, coma, and death. Therefore, the disease requires life long treatment with exogenous insulin for survival, an alternative could be whole pancreas or islet cell transplantation.[10]

## Age of onset

The rate of β cell destruction varies from patient to patient, but tends to be more aggressive in infants and adolescents of 10-14 years of age group, i.e., pubertal age group. Hence, type 1 diabetes usually presents during childhood or adolescence, although it may develop much later in life, even in the 8th and 9th decades of life. The variation in age at onset could be indicative of disease heterogeneity, with different mechanisms leading to β cell destruction in childhood onset versus adult onset diabetes.[11]

It has been observed that the onset of type 1 diabetes in younger children may be associated with a much stronger influence of DRB1*0301/DRB1*04 and INS I/I genotypes, while the development of type 1 diabetes in older children is especially influenced by the combination of INS I/I and CTLA4 G/G homozygosity. It is suggested that combined affect of INSI/I and CTLA4G/G genotype in older children with type 1 diabetes is related to time of puberty as during puberty immune response and hormonal requirements are at high levels.[12] The explanation for children having the combined INS I/I and CTLA4 G/G genotype, develops type 1 diabetes at a later age of onset involve insulin requirements and the immune response, both of which are at their peak during puberty. Thus, any mechanism resulting in reduced insulin secretion and/or inhibition of the immune response would be likely to result in type 1 diabetes. The experimental observation is that, most of the cases class III alleles are coupled with higher levels of *INS* transcription than class I alleles in the thymus. The higher *INS* expression may more efficiently induce tolerance to insulin.[13]

## Screening for type 1 diabetes in presumably healthy individuals

Type 1 diabetes usually being an autoimmune disease, characterized by the presence of a variety of autoantibodies to protein epitopes on the surface of or within the β cell of the pancreas. The presence of such markers before the development of overt disease can identify patients at risk.[14] Markers of the immune destruction of the β cell include ICAs, IAAs, GAD65, and autoantibodies to the IA-2 and IA-2- β.[15,16] Therefore, those with more than one autoantibody (i.e., ICA, IAA, GAD, IA-2, and TTG) are at high risk.[12] During initial detection of fasting hyperglycemia, 85-90% of individuals shows presence of one or more of these autoantibodies. Also, the disease has strong HLA associations, with linkage to the DQA and B genes, and also it is influenced by the DRB genes.[17–20]

Recently a new autoantibody zinc transporter isoform 8(ZnT8) has shown strong association with type 1 diabetes. The results point out that variant residue at amino acid 325 is a key determinant of humoral autoreactivity to zinc transporter isoform 8 (SLC30A8). Type 1 diabetes is associated linked to stress responses and ZnT8 causes changes in the secretory pathway, which lead to cell apoptosis and thus directly to reduction of β cells mass or activation of underlying autoimmunity.[21,22]

These HLA-DR/DQ alleles can be either predisposing or protective. In this form of diabetes, the rate of β cell destruction is quite variable, being rapid in some individuals (mainly infants and children) and slow in others (mainly adults). The protective haplotype is HLA-DR2: With 0102, 0602, and 1501. Main predisposing haplotypes in Caucasians (95%) are:HLA-DR2: With 0102, 0502 and 1601;HLA-DR3: also associated with Xp11 locus; and HLA-DR4: With 0301, 0302 and 0401 or 0402.[23–25] Many studies have implicated HLA-DQ molecules, especially DQ8 and DQ2, in governing susceptibility to type 1 diabetes, whereas DQ6 is associated with protection.[26]

At present, however, many reasons preclude the recommendation to test individuals routinely for the presence of any of the immune markers outside of a clinical trials setting. First, cut-off values for some of the assays for immune markers have not been completely established for clinical settings. Second, there is no consensus yet as to what action should be taken when a positive autoantibody test is obtained. Though, recent advancement in anti-CD3 monoclonal antibodies and GAD vaccination has given some measures that might prevent or delay the clinical onset of disease. The trial of alum-formulated GAD (GAD-alum) treatment in recent onset of type 1 diabetes patients showed that it does preservation of residual insulin secretion, although it did not change the insulin requirement.[27–29]

## Incidence

The incidence of type 1 diabetes has shown remarkable variations between ethnic groups: higher risks among Caucasians (with the highest incidence rates found in northern Europe), less in Blacks and extremely low in Asians.[30] These inter-ethnic differences are said to point to a genetically determined susceptibility in the incidence of type 1 diabetes. The incidence rate of type 1 diabetes occurring below the age of 15 years varies considerably across the world. In Caucasian populations, including those in Northern Europe, type 1 diabetes incidence rates are high with rates in excess of 20 cases/year/100,000 individuals. In contrast, countries in Asia have extremely low type 1 diabetes incidence rates, less than 1 case/year/100,000 individuals. This low incidence rate in the Asian population may be related to the population frequency distribution of susceptible.[31]

## Indian Perspective

The early work on type 1 diabetes was done by Ahuja. In his work he mentioned that in Asia type 1 diabetes is of low frequency.[32]

The incidence of type 1 diabetes from Madras was reported to be 10.5/100,000 per year. Also another study which was conducted for about ten years in South Indian based diabetes group suggested that the frequency of type 1 diabetes is around 2-5 % of diabetes.[33,34]

Many studies on Indians living abroad did show that Asian Indian children, when exposed to adverse environmental factors developed type 1 diabetes as much as the white Caucasian children do.[35]

The problem of epidemiological study in India is that the studies till date is going on in Hospitals, that too single hospitals. The data is from regions, but not as Indian data.

The previous studies showed that in India, the prevalence of type 1 diabetes varies from about 1.6 to 10.1 per hundred thousand.[35,36] The epidemiological study conducted in South Indian population for a period of four years, indicated that the prevalence of type 1 diabetes in India is increasing. The prevalence of type 1 diabetes in India is 10.1-10.6 per hundred thousand.[37] In view of lack of effective monitoring mechanism, it is not possible to know exact number of sufferers.

## Susceptibility determinants of type 1 diabetes

The disease runs in families, with a 6% risk that a sibling will develop the disease, compared with a 0.4% risk in the normal population. However, the pattern of inheritance is complex. Twin studies suggest that approximately one third of the disease susceptibility is genetic. Several loci are involved, and it is possible that the heterozygote has an increased susceptibility compared to the homozygote on some of the loci. Increased concordance for type1 diabetes in MZ twins (30%-50%), compared to that in DZ twins (4.8%-27%) and in siblings (4.4%-12.5%), suggests that susceptibility is determined partly by genetic factors, although the relatively low concordance rate among identical twins suggests that the susceptibility genes have low penetrance; that is, not all individuals who are genetically “at risk” of type 1 diabetes will develop the disease. Discordance between identical twins may reflect the generation of disparate immunological repertoires, through random rearrangement of the genes encoding T-cell receptors and β cell receptors, stochastic events, or somatic mutations.[38–44]

## Environmental factors

Epidemiological evidence supports the role of environmental factors in the development of human type 1 diabetes. An epidemiological relationship between viral infections and type 1 diabetes has been documented. Environmental factors modify the incidence and prevalence of diabetes in animal models.[45]

Environmental factors include viral infections; dietary factors in early infancy, vaccination, climatic influences, toxins, and stress. Certain viruses have been associated with β cell destruction. Type 1 diabetes occurs in patients with congenital rubella,[46] although most of these patients have HLA and immune markers characteristic of type 1 diabetes. In addition, coxsackievirus B, cytomegalovirus, adenovirus, and mumps have been implicated in inducing certain cases of the disease. It is generally believed that the environmental agents trigger disease development in genetically susceptible individuals.[47–49]

Several population studies have found a link between exposure to cow's milk and increased risk for type 1 diabetes in genetically susceptible individuals. A few studies have also shown an increased risk for infants exposed to cow's milk or cow's based formulas within three months, and also in later life. It has been found that infants fed cows milk had increased levels of bovine insulin anti-bodies compared to bovine insulin appear to cross-react with human insulin. Bovine insulin is considered immunogenic because it differs from human insulin by three amino acids.[50]

It is shown that mouse models develop type 1 diabetes following repeated injections of low-dose streptozotocin, an agent with selective toxicity to β cells. Evidence that islet autoantigens, including GAD or the p69 antigen, share cross-reactive determinants with known pathogens (e.g. Coxsackie virus B4) or exogenous antigens BSA (bovine serum albumin) raise the possibility that common environmental factors can be involved in breaking self tolerance. A direct interaction with islet β cells, as in the case of streptozotocin or Coxsackie virus B4, may alter the antigenicity of islet autoantigens. An anti β cell response can be elicited by cross-reactive antigens (molecular mimicry). Alternatively, the interaction with lymphoid or antigen presenting cells as in the case of viral infections are proposed to explain disease acceleration or protection possibly conferred by environmental factors.[51] However, recent observations suggest a more complex model in which exposure to multiple environmental factors throughout life influences the penetrance and expression of genetically determined immune dysregulation. This model is supported by the observation that multiple infections during the first few years of life are associated with a decreased risk of developing type 1 diabetes, whereas an increased risk is associated with perinatal infections. This suggests that environmental factors may modify the developing immune system in an age dependent manner and may therefore promote or attenuate disease at different stages of development, depending upon the timing and number of exposures.[11]

Increased levels of picornavirus named Ljungan virus (LV) antibodies in newly diagnosed type 1 diabetes patients show a possible zoonotic relationship between Ljungan virus infection and human type 1 diabetes.[52] It has been observed in bank voles *(Clethrionomys glareolus)* kept in captivity develop diabetes mellitus to a significant extent. A LV has been found in the pancreas of these bank voles. Moreover, LV infections in combination with environmental factors cause glucose intolerance/diabetes in normal mice.[53] The work demonstrates that diabetes-like disease can be virus-induced in a mouse model. Early in utero viral insults can set the stage for disease occurring during adult life, but the final manifestation of diabetes is dependent on the combination of early viral exposure and stress in adult life.[54]

## Gene - environment interactions

Most postulated mechanisms for environmental effects on the risk of type 1 diabetes involve the initiation of disease; either due to cross reactivity of immune responses to food or microorganisms to β cell constituents, or through tissue tropism and cytotoxicity of infectious agents, resulting in antigen shedding and priming of an autoimmune response.

Several infections might have to act in concert to precipitate clinical autoimmunity and in some infections viruses may play a role in prevention rather than precipitation of disease. A molecular constituent of *Mycobacterium bovis* cell wall (mycolylarabino-galactanpeptidoglycan; MAPG) capable of preventing diabetes in nonobese diabetic (NOD) mice. This appears to be the molecular basis of the diabetes protection attributed to complete Freund's adjuvant (CFA) and whole *Mycobacterium bovis* in the model. In contrast, exposure of intact skin to bacterial robosylating exotoxin can exacerbate disease in NOD mice. Again, the effect appears to be a modulation of on-going inflammation, rather than due to conventional antigen priming.[55]

The human intestinal microbial environment also influences type 1 diabetes pathogenesis. The experiments on NOD mice has shown that it can be affected by the microbial environment in the animal housing facility or by exposure to microbial stimuli, such as injection with mycobacteria or various microbial products. Till date the work has been done on NOD mice. The knowledge-based use of live microbial lineages or microbial products could present new therapeutic options for disease's future perspective.[56]

An RNA helicase named IFIH1 is also involved in the innate immune response to viral infection as a risk factor for type 1 diabetes. IFIH1 is located on 2q24 (IDDM19)[57]

## Risk Factors

There have been two ideas in developing type 1 diabetes autoimmune diseases -

- One is to directly attack the β cell,
- Another by affecting the developing immune system thus interfering with the future self/non-self discrimination capacity.

This latter idea has been elaborated by immunologists based on experimental studies suggesting that infrequent infections and more widespread use of vaccinations lead to an increased risk of both atopic disease and childhood diabetes.[58] Vaccinations have been investigated as possible modulators of the risk of childhood diabetes. The findings has given mixed results, hence it is inconsistent, like no detectable effect on incidence of type 1 diabetes was reported after removal of either Bacille Calmette-Guerin (BCG) or pertussis from the Swedish national immunization programme, where as the elimination of mumps by a vaccination programme in Finland has been linked to the subsequent arrest of the incidence increase in diabetes among children aged 5-9 years, though the incidence among children aged 0-4 years has continued to rise.[59–61]

BCG vaccination has an immune-modulatory role and is associated with decreased autoantibody positivity in South Indian diabetic patients.[62]

## Biomarkers

The autoimmune etiology of type 1 diabetes is also reflected by the presence of circulating autoantibodies, specific for β cell proteins including insulin (IAAs), 65-kDa isoform of GAD-1 and insulinoma-associated protein-2 (IA-2).[63,64]

IA-2, a member of the protein tyrosine phosphatase family; is a major auto antigen in type 1 diabetes.[65] IA-2 antibodies are more prevalent in patients with younger age of disease onset and in patients with diabetes susceptibility with HLA-DR4 alleles.[66] IA-2 antibodies are detected in 60–70% of type 1 diabetic patients and are associated with rapid progression to diabetes in relatives of patients.[67,68]

These autoantibodies are detectable in 85–90% of subjects with diabetes at the time of diagnosis. The appearance of the autoantibodies precedes the clinical onset of disease, often by several years. The other established autoantibodies are islet cell autoantibodies (ICAs), a protein tyrosine phosphatase with unknown function in the islet cell.[69–71] ICA has been found in the majority of type 1 diabetics of recent onset, with the percentage falling rapidly thereafter, and is associated with an increased incidence of the HLA B-8 haplotype.[65] GAD65 and IA-2 antibodies are present in approximately 26% cases of type 1 diabetes in North India.[58]

The frequency of GAD65 and ICA512 antibodies in Indian type 1 diabetes patients is similar to that in Caucasians.[72,73]

However, in recent onset of type 1 diabetes patients reports lower frequency of ICA than that reported in White Caucasian populations. These variations in ICA frequency could, in part, be related to differences in HLA associations between North Indian and White Caucasians.[74]

The presence of antibodies against an as yet unidentified 38-kDa Glycated islet cell membrane associated (GLIMA) protein has been proposed as a new biological marker of pre-clinical and recent-onset type 1 diabetes.[66,67]

Antibodies to the 64,000 M_r_ proteins are, however, the earliest and most reliable predictive marker of type 1 diabetes. GAD65 is reported that the 64,000 M_r_ Islet cell autoantigen is a form of glutamate decarboxylase, the enzyme responsible for the synthesis of γ-amino butyric acid (GABA) in brain, peripheral neurons, pancreas, and other organs. The GAD2 gene encodes GAD65 shares ten identities and nine similarities with the P2-C protein of coxsackievirus, an agent often suggested arising by ‘molecular mimicry’ between GAD and a viral polypeptide.[69,75–77]

Minor marker studies also include the Tissue Transglutaminase Antibody (TTG-Ab). The TTG is an 85-kDA protein. It is intracellular enzymes released from cells during wounding and belongs to family of calcium dependent enzymes that catalyze cross-linking of proteins. The study reports from India shows that type 1 diabetes patients have a significantly high proportion of TTG-Ab compared to healthy controls.[19]

## Genetic factors

The genetic determinants of susceptibility to type 1 diabetes are better understood than the environmental risk factors. The first diabetes susceptibility genes to be identified were the human leukocyte antigen (HLA) genes, located on chromosome 6p21.31. Subsequent studies demonstrated an association between the disease and the insulin gene region on chromosome 11p15.5. About 18 regions of the genome have been linked with influencing type 1 diabetes risk. These regions, each of which may contain several genes, have been labeled IDDM1 to IDDM18. The best studied is IDDM1, which contains the HLA genes that encode immune response proteins. There are other non-HLA genes, which have been identified. One of these non-HLA genes, IDDM-2, is the insulin gene, and the other non-HLA gene IDDM-3, maps close to CTLA4, which has a regulatory role in the immune response (chromosome 15q26).[78,79] Also, PTPN22 (protein tyrosine phosphatase, non-receptor type 22), and the regions around the interleukin 2 receptor alpha (IL2RA/CD25) and interferon-induced helicase 1 genes shows association with type 1 diabetes.[25] The defined and putative genetic factors for type 1 diabetes are presented in Table 1.

**Table 1:** Defined and putative genetic factors for type 1 diabetes[80]

Locus	Chromosome	Candidate genes	Markers	LOD	λS
IDDM1	6p21.31	HLA DR/DQ	TNFA	116.38	3.35
IDDM2	11p15.5	Insulin VNTR	D11S922	1.87	1.16
IDDM3	15q26	D15S107	D15S107	NR	NR
IDDM4	11q3.3	MDU1, ZFM1, RT6, ICE, LRP5, FADD, CD3	FGF3, D11S1917	NR	NR
IDDM5	6q25	SUMO4, MnSOD	ESR, a046Xa9	NR	NR
IDDM6	18q21-21	JK(Kidd), ZNF236	D18S487, D18S64	NR	NR
IDDM7	2q31-33	NEUROD	D2S152, D251391	3.34	1.19
IDDM8	6q25-27		D6S-281,-264,-446	NR	NR
IDDM9	3q21–25		D3S1303, D10S193	NR	NR
IDDM10	10p11–q11		D10S565	3.21	1.12
IDDM11	14q24.3–q31	ENSA, SEL-1L	D14S67	NR	NR
IDDM12	2q33	CTLA-4	(AT)n3'UTR, A/G, Exon1	3.34	1.19
IDDM13	2q34	IGFBP2, IGFBP5, NEUROD, HOXD8	D2S137, D2S164, D2S1471	NR	NR
IDDM15	6q21		D6S-283,-434,-1580	NR	NR
IDDM16	14q32	IGH		NR	NR
IDDM17	10q25.1		D10S1750.D10S1773	NR	NR
IDDM18	5q31.1–33.1	IL-12B	IL-12B	NR	NR
IDDM19	2q24.3	IFIH1		NR	NR
	1q42		D1S1617	NR	NR
	16p12-q11.1		D16S3131	1.88	1.17
	16q22-q24		D16S504	2.64	1.19
	17q25			NR	NR
	19q11			NR	NR
	3p13-p14		D3S1261	1.52	1.15
	9q33-q34		D9S260	2.20	1.13
	12q14-q12		D12S375	1.66	1.10
	19p13.3-p.13.2		INSR	1.92	1.15
PTPN22	1p13	PTPN22(LYP)	SNP=R620W	NR	1.05
SUMO4	6q25 (IDDM5)	SUMO4	SNP=M55VA allele163[G]	NR	NR

### Human Leucocyte Antigen (HLA) Genes

An association between HLA class I alleles and type 1 diabetes were first discovered by serological typing in the early 1970s.[80,81] Subsequently, a closer association was demonstrated between HLA DR3 and DR4 alleles with the type 1 diabetes.[82] The major histocompatibility complex (MHC) region on chromosome 6p21.31 (IDDM 1) is a major susceptibility locus for the common mulitfactorial form of type 1 diabetes, estimated to account for 40%-50% of λs. The MHC spans a 3.5 Mb region of chromosome 6p21.31 and consists of over 200 genes arranged into three sub regions, class I, class II, and class III.[83]

In Caucasians, the HLA-DQ heterodimers encoded by the DQA1*0301, DQB1*0302 and DQA1*0501, DQB1*0201 alleles have the strongest association with type 1 diabetes.[84] These alleles are in linkage disequilibrium with the HLA-DR4 and -DR3 alleles. In these populations, only half of the people inherit an allele of DR gene called DR3 and DR4, and less than 3% of the people have two alleles. However, in type 1 diabetes at least one allele of DR3 or DR4 is found in 95% in Caucasians, and individuals with both DR3 and DR4 are particularly susceptible to type 1 diabetes, whereas, the DR2 allele is protective.[85] DQB1*0302 differs from DQB1*0301 at position 57, where it lacks an aspartic acid residue. Sequences in the DQB1 gene that code for an amino acid other than aspartic acid at position 57 (non-ASP57) are highly associated with type 1 diabetes.[86] It is suggested that the amino acid residue at position 57 of the DQ-β chain appears to be critical for peptide binding and recognition.[87] The diabetes risk of non-ASP57 is further increased when the haplotype also contains the DRB1*0401 allele, suggesting the possible existence of at least two separate loci of susceptibility.[88] Almost all studies are consistent in demonstrating positive associations between type 1 diabetes and DRB1*0401-DQA1*0301-DQB1*0302 and DRB1*0301-DQA1*0501-DQB1*0201 haplotypes, with the strongest association seen in the heterozygote for the high-risk haplotype. In addition to these two high-risk HLA haplotypes, there are other less common HLA haplotypes associated with high diabetes risk in different population[87,89–93] [Table 2].

**Table 2:** Distribution of HLA Class II alleles across different population[89–104]

Population	Susceptibility	Protective
Caucasians	DQA1*0301,	HLA-DR2=DRB1*1501–DQA1*0102–DQB1*0602
	DR3= DRB1*0301-DQB1*0201	
	DR4=DRB1*0401-DQB1*0302	
	DQA1*0501	
	HLADRB1*0301–DQA1*0501–DQB1*0201	
	DQB1*0201	
	A1-B8-DRB1*03-DQB1*02	
Spanish	DQA1*0101-DQB1*0501-TNFa2b1	TNFa10b4, DQA1*0501-DQB1*0301
	DQA1*0201-DQB1*0202- BAT-2*2	DQA1*0103-DQB1*0603
Sardinia	DRB1*0301(DR3)–DQA1 *0501 -DQB1*0201	HLA-DR2=DRB1*1501–DQA1*0102–DQB1*0602
	DRB1*0401	
	DRB1*0405	
Finnish[93]	DRB1*0405-DQB1*0302	DQB1*0602 or *0603
	DRB*0401-DQB1*0302	
	DRB*0404-DQB1*0302	
	DRB1*04-DQB1*0304	
	(DR3)-DQA1*05-DQB1*02	
Swedish[90]	DQA1*0301/DQB1*0302	DR15-DQA1*0102-DQB1*0602
	DQA1*0301/DQB1*0302 and DQA1*0201/DQB1*0501	
Belgian[89]	DR4-DQ8=HLA DRB1*04-DQA1*0301-DQB1*0302	DRB1*0403
	DR3-DQ2=DRB1*03-DQA1*0501-DQB1*0201	
Yemenite jews[92]	DRB1*03011,*0402	HLA-DR2 and DQB1*0602
Blacks	DR3=DRB1*0301-DQB1*0201	DRB1*1501–DQA1*0102–DQB1*0602
	DRB1*0401	DRB1*1503–DQA1*0102–DQB1*0602
	DRB1*0405	DRB1*1501–DQA1*0102–DQB1*0602
Asians[91]	DQB1*0302	DRB1*1501–DQA1*0102–DQB1*0602
	DR4=DRB1*0405-DQB1*0401	
Japanese[95]		
	DQB1*0201	
	HLA-DR2=DRB1*1501–DQA1*0102–DQB1*0602	
	DRB1*0405–DQA1*0301–DQB1*0401	DRB1*1502–DQA1*0103–DQB1*0601
	DRB1*0901–DQA1*0301–DQB1*0303	DRB1*0405–DQB1*0401
	DQB1*0402	
	DR4=DRB1*0405–DQB1*0401	DRB1*0901–DQB1*0303
	DR9=DRB1*0901–DQB1*0303	
Korean[94]	DR4=DRB1*0405–DQB1*0401	DR15-DQB1*0602
	DR9=DRB1*0901-DQB1*0303	DR11/12-DQB1*0301
	DR3=DRB1*0301-DQB1*0201	
	DQ2 =DQA*0501–DQB*0201	
Indian[96–104]	DRB1*0301	
	DRB1*0401, DRB1*0405	DRB1*0403, DRB1*0404
	DQB1*0201	DRB1*1501-DQBr0602
	DR3=DRB1*0301–DQB1*0201	DRB1*0701
	DQ2 =DQA*0501–DQB*0201	
	A26-B8-DRB1*03-DQB1 *02	
	Ax-B50-DRB1*03-DQB1*02	

Type 1 diabetes is supposed to be less frequent in Asians than in Caucasians. It is suggested that low incidence of type 1 diabetes in Asians are due to genes, which protect against type 1 diabetes. In general, in Asians, it is very common that the protective DR4 is associated with the susceptible DQ alleles while the neutral/protective DQ allele is associated with the susceptible DR4 alleles. This counterbalancing influence between susceptible DRB1 and protective DQB1, and vice versa, might be an important factor responsible for the low incidence of type 1 diabetes in Asians. In Asians, in addition to DQB1*0302, DQB1*0401 on DR4 haplotypes is positively associated with type 1 diabetes. This contribution of the DQ molecules to overall disease susceptibility might be genotype dependent and/or may be influenced by the DRB1*04 allele on the haplotype.[95]

The homozygosity and heterozygosity of DRB1*0301 and DRB1*04 alleles significantly associated with type 1 diabetes in North India.[96–98] Significant increase of DRB1*0301 and DQB1*0201 in the patients is observed in North Indian studies. It is also observed that DRB1*0401 and DRB1*0405 is significantly increased and DRB1*0403 and DRB1*0404 to be significantly decreased in the patients as compared to controls. The studies demonstrate that DR3 and DQ2 are positively associated with type 1 diabetes.[26,98] The Indian samples show HLA-C*0702 allele whereas the European 8.1AH has the HLA-C*0701 allele. The Indian samples also differs from the European 8.1AH at DRB3 in that they had DRB3*02 whereas the European 8.1AH has DRB3*0101. The Indian samples shared HLA-B*0801 and DQB1*02 with the European 8.1AH.[99–102]

Molecular analysis of DR4 sub typing revealed the heterogeneous prevalence of the DR4 subtypes in the type 1 diabetes patient group. Distribution of DR4 alleles in the healthy Indian population has revealed the presence of *0403 as the most frequent allele, followed by *0404, *0405, *0401, * 0406, and *0412 in descending order of frequency.[103]

It is observed that the DRB1*0403 frequency is more in the Japanese, but rarely in Caucasians. The distribution of HLA-A*02 subtypes suggest that Asian Indians have shared parallel natural selection driving forces and racial admixing with the Oriental populations, which has led to analogous influence on HLA diversity.[104]

In the North Indian study reported that the haplotype A26-B8-DR3 as the most common autoimmunity-favoring haplotype encountered among the patients. This association is, indeed, unique to Indian autoimmune patients, as it replaces the otherwise most commonly associated Caucasian haplotype A1-B8- DR3 (AH8.1) in this population.[26,104]

### Major Histocompatibility Complex Class I Chain-Related Gene- A (MICA)

The MICA – MICE genes, members of the MIC (major histocompatibility complex (MHC) class I chain-related) family are spread across the 2-Mb MHC class I region.[105] The MICA gene is the nearest neighbor to HLA-B (only 46 kb centromeric). In addition, the expression of MICA gene is induced by heat shock and is broadly recognized by a subset of γ§ T-cell that exists predominantly in the intestinal and other epithelia.[106] However, the broad recognition of MICA and MICB implies an unusual mode of interaction with the natural killer cells and Vd1 γ§T-cell, and the generality of this system is not decisively proven. Significant evidence is there for the association between MICA5.1 and adult-onset type 1 diabetes.[100,105,107]

It is observed that frequency of A5.1 allele is highest in early-onset patients, intermediate in intermediate-onset patients and lowest in late-onset patients. A5.1 allele is strongly associated with HLA-B7 and Cw7, suggesting that MICA*A5.1-B7-Cw7 haplotype contains a gene responsible for age-at-onset. A4 allele was associated with a susceptible haplotype, DR4-DQB1*0401, and A6 allele is associated with a protective haplotype, DR2-DQB1*0601, suggesting that the association of MICA with type 1 diabetes susceptibility may be due to linkage disequilibrium with class II haplotypes.[107,108]

The microsatellite polymorphism consists of repetitions of GCT/AGC. (GCT/AGC)n encodes polyalanine, and the number of alanine residues differs with the number of triplet repeats. A5.1 allele contains five triplet repeats plus one additional nucleotide insertion (GGCT/AGCC), causing a frameshift mutation resulting in premature termination by the stop codon (TAA) in the transmembrane (TM) region. The MICA protein encoded by A5.1 allele influences the activation of NK cells, which modify β cells destruction and thus involve in the age-at-onset of type 1 diabetes.[107,109]

Meta-analysis has shown that the MICA*A5 variant was significantly associated with an increased risk for type 1 diabetes, while MICA*A6 was significantly associated with a decreased risk. Depending upon with which risked allele MICA*A6 is present, MICA*A6 confers susceptibility or protection. For example, MICA alleles when present with type 1 diabetes-associated high-risk MHC class II haplotypes (HLA DQ2DR17) revealed that MICA*A6 was associated with an increased risk for type 1 diabetes. In contrast, MICA*A6 reduced the risk from the HLA DQ8DR4 type 1 diabetes-risk haplotype. MICA*A9 showed when association with DQ8DR4 haplotypes showed to increased risk for type 1 diabetes.[110,111]

### Insulin (INS) Gene

Susceptibility to type 1 diabetes by 5' regulatory region of the insulin (INS) gene on chromosome 11p15.5 (IDDM2) is 10%. Insulin is synthesized by the β cell of the islets of Langerhan's in the pancreas and is secreted into the bloodstream in response to a number of stimuli, the most important of which is serum glucose levels.[112] Insulin exerts its action in cells by binding to a tyrosine kinase-coupled receptor on the cell surface. Insulin receptor substrate (IRS) protein is phosphorylated on its tyrosine residue; leading to the activation of a cascade of proteins in multiple downstream signaling pathways mediating the metabolic actions of insulin. Insulin plays a key role in glucose homeostasis alongside a counter regulatory hormone, glucagon, which raises serum glucose.[112] IDDM2 consists of a highly polymorphic stretch of 14–15 bp repeats of DNA lying 365 bp upstream of the initiation of transcription of the INS gene.[113] Because the repeated sequences follow one behind the other (in tandem) and because the number repeats varies between individuals, this phenomenon is called variable number of tandem repeats (VNTRs).[96,114] There are three classes of VNTRs in the insulin gene: (i) Class I (26-63 repeats), (ii) Class II (approximately 80 repeats), and (iii) Class III (40-200repeats).[115]

The class I VNTRs is most common in Caucasians; with around 70% of alleles being in the range of 30-44 repeats. The class II alleles are rare. Homozygosity class I VNTR determines high risk for diabetes, while class III VNTRs confers dominant protection. The VNTR class III allele is associated with higher expression of messenger RNA (m-RNA) for insulin within the thymus.[112,114] The presence of at least one class III allele is associated with a 3-fold reduction in the risk of type 1 diabetes.[114,115] One hypothesis is that expression of insulin within the thymus leads to negative selection (deletion) of auto reactive T-cell and thus to development of tolerance.[115,116] VNTR class I alleles, associated with low levels of proinsulin and insulin in the thymus during fetal life and childhood, may fail to shape the T-cell repertoire and lead to the presence of anti-insulin auto reactive T cell.[117,118]

### Cytotoxic T Lymphocytes Associated Antigen 4(CTLA 4) Gene

The IDDM12 is mapped to CTLA4 gene, mapped to chromosomes 2q33.[119] CTLA4 protein is a member of the immunoglobulin superfamily and is a costimulatory molecule expressed by activated T cells. CTLA4 transmits an inhibitory signal to T cells^51^. It is present in exon 1, codon 17 position, giving Threonine (Thr) to Alanine (Ala), substitution in the leader peptide of the protein. Meta-analysis studies also revealed that the CTLA-4*G genotype is associated with type 1 diabetes.[120] Several population studies showed increased transmission of the G allele of a 49A->G polymorphism associated with type 1 diabetes.[121] Asian studies yielded significant and reliable linkage evidence for the susceptibility of type 1 diabetes.[11,31,119] Japanese study results showed a significant correlation between CTLA4 gene polymorphism and ICA 512 Ab.[122]

### Small Ubiquitin-Related Modifier (SUMO4) Gene

Studies in IDDM 5 have lead to the discovery of a novel polymorphism 163 Alanine (A)->Glycine (G), of SUMO4 gene, associated with risk to type 1 diabetes in Asians, but not in Caucasians. Also, no association is observed with SUMO4 M55V and type 1 diabetes in Asian-Indian patients.[123] The SUMO4 gene maps to 6q25. It is shown that SUMO4 M55V is not associated with susceptibility to type 1 diabetes by itself. The observation based large controls-cohort studies yields that the presence of SUMO4 GG increased further the relative risk conferred by HLA-DR3/DR4 to type 1 diabetes, whereas SUMO4 AA decreased the risk. Though, SUMO4 M55V is not associated with susceptibility to type 1 diabetes in Caucasians populations like, Latvians; it is still considered to be an important marker for type 1 diabetes and designated as IDDM 5.[124,125]

The novel polymorphism M55V, causing an amino acid change in the evolutionarily conserved met55 residue has been shown to activate the nuclear factor kappaB (NF-kappaB), hence the suspected role of SUMO4 in the pathogenicity of type 1 diabetes.[126,127]

### Genome wide screening

The Wellcome Trust Case Control Consortium (WTCCC) primary genome-wide association (GWA) scans shows through single point associations at P<5 × 10^-7^ between type 1diabetes and six chromosome regions: 12q24, 12q13, 16p13, 18p11, 12p13 and 4q27. It is confirmed that associations of 12q24, 12q13, 16p13 and 18p11 (P≤ 1.35 × 10^-9^; P≤ 1.15 × 10^-14^), leaving eight regions with small effects or false-positive associations. The study increases the number of type 1 diabetes loci with compelling evidence from six to at least ten. Further, multilocus analyses shows four regions levels of significance (chromosomes 4q27 and 12p13), and through the combined analysis of autoimmune cases two regions have been studied (chromosomes 18p11 and the 10p15 CD25 region). The associations with type 1 diabetes for chromosomes 12q13, 12q24, 16p13 and 18p11 have been confirmed in independent and multiple populations.[119,128]

The two signals on chromosome 12 (at 12q13 and 12q24) map to regions of extensive linkage disequilibrium covering more than ten genes have been reported. Several of these represent functional candidates because of their presumed roles in immune signaling, considered to be a major feature of type 1 diabetes-susceptibility. These include ERBB3 (receptor tyrosine-protein kinase erbB-3 precursor) at 12q13 and SH2B3/LNK (SH2B adaptor protein 3), TRAFD1 (TRAF-type zinc finger domain containing 1) and PTPN11 (protein tyrosine phosphatase, non-receptor type 11) at 12q24. Of those listed, PTPN11 is a particularly attractive candidate given a major role in insulin and immune signaling.[129]

Scan result shows associations with SNPs within the chromosome 10p15 region containing CD25, encoding the high-affinity receptor for IL-2. The association between type 1 diabetes and a region of 4q27 is revealed by the multilocus analysis. This region contains the genes encoding both IL-2 and IL-21. Together with studies in the NOD mouse model of type 1 diabetes, which have shown that a major non-MHC locus (IDDM3) reflects regulatory variation of the Il2 gene. In the multilocus analysis, there is increased support for a region on chromosome 12p13 containing several candidate genes, including CD69 (early T-cell activation antigen) and multiple C-type lectin domain family (CLEC) genes.[130]

A genome wide search in a Dutch isolated population showed evidence for type 1 diabetes loci on chromosome 8q24 (marker D8S1128) and on chromosome 17q24 (marker D17S2059). Both the 8q and 17q localization are supported by allele-sharing at adjacent markers in affected individuals.[119] Also, evidences of interaction between new loci like 6q21 for type 1 diabetes susceptibility were reported from Scandinavian families.[131]

A genome-wide association study of nonsynonymous SNPs identified a type 1 diabetes locus in the IFIH1 (also known as mda-5 or Helicard) present on chromosome 2q24.3region. The T1DGC 6K SNP scan and follow-up studies confirmed that type 1 diabetes associations at *INS,IFIH1* (interferon-induced helicase), and *KIAA0350* and identified an additional disease association on chromosome 21q22.3 in the *UBASH3A* locus. This gene and its flanking regions are now validated targets for further re-sequencing, genotyping, and functional studies in type 1 diabetes.[132] Studies based on large trio studies showed that the risk ratio for the minor allele of the nsSNP rs1990760 A → G (A946T) is 0.86 (95% confidence interval = 0.82–0.90) at *P* = 1.42 × 10^-10^, which is a convincing statistical support.[133]

Linkage disequlibrium (LD) mapping has become a vital tool for both confirmation and fine-mapping of susceptibility intervals, as well as identification of etiological mutations. The assessment of huge and ethnically varied data sets has allowed identification of haplotypes that differ only at a single codon in a single locus. As more data become available, the study of pairs of haplotypes which differ at a single polymorphic site, but have different effects on disease susceptibility, should allow more precise definition of the polymorphisms involved in the disease process.[31]

## Steps taken for molecular characterization of type 1 diabetes

After several years of work, type 1 diabetes is probably the best characterized of all common multigenic diseases. Of the 18 genomic loci, variation at HLA alone explains much of the risk to siblings, followed by INS and CTLA4 loci. There are other risk alleles, although their effects are weaker than is seen for HLA and INS. The pathways for this identified genes are though yet to be identified so that it will provide insight into etiology, pathogenesis, and perhaps even prevention or treatment. In addition, the exact variants that increase risk in the HLA region are unknown, in part because linkage disequilibrium makes untangling the relevant variants difficult. Also, to prevent the diabetic complications, such as diabetic retinopathy and nephropathy, the genetic constitution of such patients need to be identified for the additional risk factors. The Genetics of Kidney in Diabetes Study (GoKinD) are working on the genetic basis of diabetic kidney disease as well as other issues concerning type 1 diabetes. Extensive work has been done on the NOD mouse model of type 1 diabetes. Other types of experiments include genome wide expression analysis of relevant tissues (such as T lymphocytes). It is likely that both linkage and association will be valuable tools to identify new variants. Improvements in genotyping technology or other advances in genome study will be hopefully permit whole-genome association studies that can scan the entire genome with good power for alleles that are associated with increased risk of type 1 diabetes.

Two major trials have been conducted to try to prevent type 1 diabetes. In the United States, the Diabetes Prevention Trial (DPT-1) started in 1994 with the aim of determining whether antigen based treatment with insulin (oral and parenteral insulin treatment in relatives at high and moderate risk) would prevent or delay diabetes.[124] These treatments did not overall slow the progression to diabetes. The European nicotinamide diabetes intervention trial (ENDIT) also found no difference in protection from diabetes when participants were assigned to either oral nicotinamide or placebo treatment.[134,135]

The International Hap Map consortium also aids Genetic studies.The major impetus for this effort is the genome can be parsed into blocks of linkage disequilibrium (averaging 11-22 Kb in size) within which the vast majority of common sequence variants are correlated with each other. Further, the alleles at variants within these blocks fall into a few simple patterns or haplotype. Because there are only a few haplotypes, genotyping a few, well-chosen single nucleotide polymorphism (SNP_s_) can accurately indicate which haplotype are present. These SNP_s_ are called haplotype SNP_s_ or ht SNP_s_. A majority goal of the Hap Map consortium is to identify a set of ht SNP_s_ that will allow much of the common variation in the genome to be interrogated efficiently for association with disease. Thus, genome-wide association studies will not require typing all 10 million common variants but rather a few hundred thousand htSNP_s_ that capture most of the common variation for any particular gene.

Much of the unexplained risk of type 1 diabetes is due to environmental factors. Identification of these environmental factors will also facilitate the search for the genetic risk factors, since it would then be possible to control for the environmental exposures, and also to search for the gene-environment interactions. Most importantly, if environmental triggers could be identified, they would provide obvious targets for preventive measures in high-risk individuals.

## Molecular approaches taken for diagnosis, therapy and prevention

Animal models have shown that insulin therapy is effective in diabetes. Pilot studies have suggested that insulin therapy also delays diabetes in humans. But long term effect can not be prevented and also, total glycemic control in type 1 diabetes is not achieved. Also, the associated risks of hypoglycemia and end-organ diabetic complications linger.[136–139]

The Epidemiology and prevention of Diabetes (EURODIAB) group, Diabetes Prediction and Prevention (DIPP) group is working towards in devising a unique molecular approach, which will enable in large population based screening and will delay or prevent the disease. Collected family data are not enough for the prediction of the type 1 diabetic patients about the risk of their sibs and/or their next generation developing the disease. It has been hypothesized that if expression of diabetes-resistant MHC class II alleles on bone marrow-derived cells is sufficient to prevent diabetes, genetic engineering of autologous hematopoietic stem cells could be used to restore expression of diabetes-resistant MHC on bone marrow–derived cells. Furthermore, expression of diabetes-resistant MHC class II alleles on bone marrow–derived cells could mediate negative selection of self-reactive T cells that cause diabetes, thereby preventing disease. This approach could have significant advantages over transplantation of allogeneic bone marrow cells, because the possibility of graft-versus-host disease would be avoided. Also, it has been demonstrated that expression of diabetes-resistant MHC class II I-Aα chain molecules on hematopoietic cells following retroviral transduction of bone marrow is sufficient to prevent the occurrence of type 1 diabetes in NOD mice.[138,140]

Intervention in autoimmune type 1A-diabetes include monoclonal antibody therapies (e.g. anti-CD3, anti-CD25, anti-CD52 or anti-CD20 monoclonal antibodies), immunosuppression (e.g. calcineurin inhibitors, B7 blockade, glucocorticoids, sirolimus (rapamycin), azathioprine or mycophenolate mofetil), immunomodulatory therapies (e.g. plasmapheresis, intravenous immunoglobulin, cytokine administration, adoptive cellular gene therapy) and tolerisation interventions (e.g. autoantigen administration or avoidance, altered peptide ligand or peptide-based therapies).[141]

A Belgium study has provided proof of principle that short-term anti-T-cell antibody treatment is able to preserve residual β cell function for at least 18 months. The resultant stabilizing effect on metabolic control is expected to delay or limit chronic complications in these patients. Both xenotransplantation and stem cell therapy provide a possible road to permanent therapy. The ultimate goal is prevention of clinical disease in prediabetes condition.[142] Also, the studies Diabetes Registry Group and others in first degree family members of type 1 diabetic patients have developed the identification of individuals at very high risk of hyperglycaemia so that new immunological treatments can be tested in the prediabetic phase. Retention of β cell function in patients with type 1 diabetes is known to result in improved glycemic control and reduced hypoglycemia, retinopathy, and nephropathy.[139,143]

Advances in understanding of the autoimmune process leading to diabetes have generated interest in the potential use of immunomodulatory agents that may collectively be termed vaccines, to prevent type 1 diabetes. Vaccines may work in various ways, including changing the immune response from a destructive (e.g. Th1) to a more benign (e.g. Th2) response, inducing antigen-specific regulatory T cells, deleting autoreactive T cells, or preventing immune cell interaction. To date, most diabetes vaccine development has been in animal models, with relatively few human trials having been completed. A major finding of animal models such as the NOD mouse is that they are extremely sensitive to diabetes protection, such that many interventions that protect mice are not successful in humans. This is particularly evident for human insulin tolerance studies, including the Diabetes Prevention Trial-1, where no human protection was seen from insulin despite positive NOD results. Further challenges are posed by the need to translate protective vaccine doses in mice to effective human doses. Despite such problems, some promising human vaccine data are beginning to emerge. Recent pilot studies have suggested a beneficial effect in recent-onset human type 1 diabetes from administration of nondepleting anti-CD3 antibodies or a peptide from heat shock protein 60.[144–146]

None of the therapies attempted to date has produced long-term remissions in new-onset type 1 diabetes patients and no therapies have been shown to prevent the disease. However, with advances in understanding of basic immunology and the cellular and molecular mechanisms of tolerance induction and maintenance, successful intervention therapies are being developed. Keeping a view on convenience, safety, and long-lasting protection, vaccines remain one of the most promising strategies to prevent type 1 diabetes.

Also, by registering as many new type 1 diabetes cases as possible within a defined region, the documents could help in knowing the correct etiology which may relate to differences in exposure to aetiological environmental triggers and in genetic background. The systematic study of clinical, biological and epidemiological data in diabetic patients, as compared to nondiabetic control subjects, is emerging as an important tool to identify putative aetiological factors or confirm their importance suggested by complementary approaches such as genetic linkage studies or animal models. Registry based association studies have also unveiled a marked age dependent heterogeneity at disease onset regarding severity of clinical presentation and the nature of the immune and genetic markers present.

The Indian population comprising of more than 30,000 estimated endogamous communities, with genomic foot prints of earliest [60 to 70,000 years before present(ybp)] out of Africa migration, inhabiting areas with climatic variations from 0° to 50° C and altitude ranging from sea level to 5,000 feet; pursuing occupations ranging from hunting gathering, pastoralism, agriculture to modern city based; and varied life styles and food habits, are perfect experimental setting and material for basic research and development leading to discovery. However, the way the effort is organized in the country, either in the public or private sector, no model is visible.

We would like to propose the following broad ‘Model’ as operational strategy:

- There has to be a conscious awareness program initiated with vigorous campaign at all India bases, the importance of Genetics and Health and the orientation must be community genetics rather than the existing patient/hospital based approach.
- The private players who are involved in RandD need to support this with adequate resources by employing professional communication experts. This is also in line with their long-term interests in terms sensitizing the Indian populations at all levels for future genome based medical practices.
- There is an urgent need to the formation of regional monitoring cells in the country for genetic diseases.

Type 1 diabetes could be a unit within these cells. Each unit can have sub-units based on the regional coverage. These units/sub T units will have trained medical and anthropological investigators, in recruiting families for monitoring. Funds need to come for this exercise from both public (ICMR/DBT/AnSI) and private partnership. The modern field level laboratory network of The Anthropological Survey of India, could form the infrastructure with all cold storage and UPS systems in place.

The monitoring which includes recruiting families for long-term evaluation with adequate resources for immediate medical intervention can form a basis for building cohorts of patients/controls from communities (with proper phenotype-environmental/life style data) for Research and Development.

An apex committee comprising members from ICMR/DBT/AnSI and private companies should resolve all issues with respect to funding these monitoring units, besides resolving ethical issues and benefit sharing mechanisms.

The usual approach of spending resources on creating structures in terms of buildings, furniture and recruiting permanent manpower should be avoided. What is important is implementing an action plan with existing structures and manpower on contract. A centralized think tank with complete de-centralized, meticulously planned field operation with quantifiable outcomes should be the MODEL.

## References

[1] Atkinson MA, Maclaren NK (1994). The pathogenesis of insulin dependent diabetes. N Engl J Med.

[2] American Diabetes Association (1997). The Expert Committee on the Diagnosis and Classification of Diabetes Mellitus: Report of the Expert Committee on the Diagnosis and Classification of Diabetes Mellitus. Diabetes Care.

[3] Aly AT, Ide A, Jahromi M M, Barker MJ, Fernando SM, Eisenbarth GS (2006). Extreme genetic risk for type 1A diabetes. PNAS.

[4] Imagawa A, Hanafusa T, Miyagawa J, Matsuzawa Y (2000). A novel subtype of type 1 diabetes mellitus characterized by a rapid onset and an absence of diabetes-related antibodies. NEJM.

[5] Puavilai G, Chanprasertyotin S, Sriphrapradaeng A (1999). Diagnostic criteria for diabetes mellitus and other categories of glucose intolerance: 1997 criteria by the Expert Committee on the Diagnosis and Classification of Diabetes Mellitus (ADA), 1998 WHO consultation criteria, and 1985 WHO criteria.World Health Organization. Diabetes Res Clin Pract.

[6] Kuzuya T, Nakagawa S, Satoh J, Nanjo K, Sasaki A (2002). Committee of the Japan Diabetes Society on the diagnostic criteria of diabetes mellitus. Report of the committee on the classification and diagnostic criteria of diabetes mellitus. Diabetes Res Clin Pract.

[7] Lan M S, Wasserfall C, Maclaren N K, Notkins A L (1996). IA-2, a transmembrane protein of the protein tyrosine phosphatase family, is a major autoantigen in insulin-dependent diabetes mellitus. PNAS.

[8] Motzo C, Contu Da, Congia M, Todd JA, Devoto M, Cucca F (2004). Heterogeneity in the Magnitude of the Insulin Gene Effect on HLA Risk in type 1 Diabetes. Diabetes.

[9] Kukreja A, Cost G, Marker J, Wilson B, Porcelli S, Maclaren N (2002). Multiple immuno-regulatory defects in type-1 diabetes. J. Clin Invest.

[10] Solimena M (1998). Vesicular autoantigens of type 1 diabetes. Diabetes Metab Rev.

[11] Ikegami H, Awata T, Kawasaki E, Kobayashi T, Maruyama T, Ogihara T (2006). The association of CTLA4 polymorphism with type 1 diabetes is concentrated in patients complicated with autoimmune thyroid disease:a multicenter collaborative study in Japan. J Clin Endocrinol Metab.

[12] Felner EI, Klitz W, Ham M, Lazaro AM, Stastnye P, White PC (2005). Genetic interaction among three genomic regions creates distinct contributions to early and late-onset type 1 diabetes mellitus. Pediatric Diabetes.

[13] Awata T, Kawasaki E, Ikegami H, Tanaka S, Kanazawa Y, Katayama S (2007). Insulin gene/IDDM2 locus in Japanese Type 1 diabetes:Contribution of class I alleles and influence of class I subdivision in susceptibility to Type 1 diabetes. J Clin Endocrinol Metab.

[14] Mayrhofer M, Rabin DU, Messenger L, Standl E, Ziegler AG (1996). Value of ICA512 antibodies for prediction and diagnosis of type 1-diabetes. Exp Clin Endocrinol Diab.

[15] Baekkeskov S, Neilsen JH, Marner B, Bilde T, Ludvigsson J, Lernmark A (1982). Autoantibodies in newly diagnosed diabetic children with immunoprecipitate human pancreatic islet cell proteins. Nature.

[16] Atkinson MA, Maclaren NK, Riley WJ, Winter WE, Fisk DD, Spillar RP (1986). Are insulin autoantibodies markers for insulin-dependent mellitus?. Diabetes.

[17] Schmidli RS, Colman PG, Harrison LC (1994). Do glutamic acid decarboxylase antibodies improve the prediction of IDDM in first-degree relatives at risk for IDDM?. J Autoimmunity.

[18] Myers MA, Rabin DU, Rowley MJ (1995). Pancreatic islet cell cytoplasmic antibody in diabetes is represented by antibodies to islet cell antigen 512 and glutamic acid decarboxylase. Diabetes.

[19] Kanungo A, Shtavere-Brameus A, Samal KC, Sanjeevi CB Autoantibodies to tissue transglutaminase in patients from eastern India with malnutrition-modulated diabetes mellitus. Ann NY Acad Sci.

[20] Hagopian WA, Sanjeevi CB, Kockum I, Landin-Olsson M, Karlsen AE, Lemmark A (1995). Glutamate decarboxylase, insulin, and islet cell-antibodies and HLA typing to detect diabetes in a general population-based study of Swedish children. J Clin Invest.

[21] Kawasaki E, Uga M, Nakamura K, Davidson HW, Hutton JC, Eguchi K (2008). Association between anti-ZnT8 autoantibody specificities and SLC30A8 Arg325Trp variant in Japanese patients with type 1 diabetes. Diabetologia.

[22] Wenzlau JM, Hutton JC, Davidson HW (2008). New antigenic targets in type 1 diabetes. Curr Opin Endocrinol Diabetes Obes.

[23] Yu L, Cuthbertson DD, Maclaren N, Jackson R, Krischer JP (2001). DPT-1 Participating Investigators Expression of GAD65 and islet cell antibody (ICA512) autoantibodies among cytoplasmic ICA+ relatives is associated with eligibility for the Diabetes Prevention Trial-Type 1. Diabetes.

[24] Huang W, Connor E, DelaRosa T, Muir A, Schatz D, Maclaren NK (1996). Although DR3-DQB1* 0201may be associated with multiple component diseases of the autoimmune polyglandular syndromes, the human leukocyte antigen DR4-DQB1* 0302 haplotype is implicated only in β cell autoimmunity. J Clin Endocrinol Metab.

[25] http://www.uchsc.edu/misc/diabetes/oxch7.html.

[26] Kanga U, Vaidyanathan B, Jaini R, Menon PSN, Mehra NK (2004). HLA haplotypes associated with type 1diabetes mellitus in North Indian children. Human Immunology.

[27] Ludvigsson J, Faresjö M, Hjorth M, Ortqvist E, Zerhouni P, Casas R (2008). GAD treatment and insulin secretion in recent-onset type 1 diabetes. NEJM.

[28] Ludvigsson J (2007). Immune intervention at diagnosis--should we treat children to preserve β cell function?. Pediatr Diabetes.

[29] Faustman D.L (2008). Immunotherapy on Trial for New-Onset type 1 Diabetes. NEJM.

[30] Serranos-Rios M, Goday A, Martienz L (1999). Migrant Populations and the incidence of Type 1 diabetes mellitus:an overview of the literature in Latin America. Diabetes Metab Res Rev.

[31] Park Y, Eisenbarth GS (2001). Genetic susceptibility factors of type 1diabetes in Asians. Diabetes Metab Res Rev.

[32] Karvonen M, Viik-Kajander M, Moltchanova E, Libman I, LaPorte RE, Tuomilehto J (2000). The incidence of type 1 diabetes worldwide – the analysis of the WHO DiaMond (Diabetes Mondiale) data from 50 countries. Diabetes Care.

[33] Rao PV, Ushabala P, Ahuja MMS (1991). A decade of epidemiology of diabetes. Intnl. J. Diab. Dev. Countries.

[34] Sridhar GR, Nagamani G (2002). Clinical association of autoimmune diseases with diabetes mellitus analysis from southern India. Ann. N.Y. Acad. Sci.

[35] Ramachandran A, Snehalatha C, Khader OMSA, Joseph TA, Viswanathan M (1992). Prevalence of childhood diabetes in urban population in South India. Diab Res Clin Pract.

[36] Ramachandran A, Snehalatha C, Sasikala R, Satyavani K, Vijay V (2000). Vascular complications in young Asian Indian patients with Type 1 diabetes mellitus. Diab Res Clin Pract.

[37] Krishna P, Roopakala, Prasanna Kumar KM (2005). Dyslipidemia in type 1 diabetes Mellitus in the young. Int. J. Diab. Dev. Countries.

[38] Degnbol B, Green A (1978). Diabetes mellitus among first- and second-degree relatives of early onset diabetics. Ann of Hum Genet.

[39] Tillil H, Kobberling J (1987). Age-corrected empirical genetic risk estimates for first-degree relatives of IDDM patients. Diabetes.

[40] Swanljung O, Meurman JH, Torkko H, Sandholm L, Kaprio E, Maenpaa J (1992). Caries and saliva in 12-18-year-old diabetics and controls. Scand J Den Res.

[41] Kumar D, Gemayel NS, Deapen D, Kapadia D, Yamashita PH, Mack TM (1993). North-American twins with IDDM. Genetic, etiological, and clinical significance of disease concordance according to age, zygosity, and the interval after diagnosis in first twin. Diabetes.

[42] Nerup J, Mandrup-Poulsen T, Helqvist S, Andersen HU, Pociot F, Lorenzen T (1994). On the pathogenesis of IDDM. Diabetologia.

[43] Kyvik KO, Green A, Beck-Nielsen H (1995). Concordance rates of insulin dependent diabetes mellitus: A population based study of young Danish twins. BMJ.

[44] Wagner A, Tibben A, Bruining GJ, Aanstoot HJ, Blondeau MJ, Niermeijer MF (1995). Preliminary experience with predictive testing for insulin-dependent diabetes mellitus. Lancet.

[45] Boitard C, Larger E, Timsit J, Sempe P, Bach JF (1994). IDDM: An islet or an immune disease?. Diabetologia.

[46] Lammi N, Karvonen M, Tuomilehto J (2005). Do microbes have a causal role in type 1 diabetes?. Med Sci Monit.

[47] King ML, Shaikh A, Bidwell D, Voller A, Banatvala JE (1983). Coxsackie-B-virus-specific IgM responses in children with insulin-dependent (juvenile-onset;type I) diabetes mellitus. Lancet.

[48] Karjalainen J, Knip M, Hyoty H, Linikki P, Ilonen J, Akerblom HK (1988). Relationship between serum insulin antibodies, islet cell antibodies and Coxsackie-B4 and mumps virus-specific antibodies at the clinical manifestation of type 1 (insulin-dependent) diabetes. Diabetologia.

[49] Pak CY, Eun HM, McArthur RG, Yoon JW (1988). Association of cytomegalovirus infection with autoimmune type 1diabetes. Lancet.

[50] Paronen J, Knip M, Savilahti E, Virtanen SM, Ilonen J, Vaarala O (2000). Effect of cow's milk exposure and maternal type 1 diabetes on cellular and humoral immunization to dietary insulin in infants at genetic risk for type 1 diabetes. Finnish Trial to Reduce IDDM in the Genetically at Risk study Group. Diabetes.

[51] Biros E, Jordan A M, Baxter GA (2005). Genes mediating environment interactions in type 1 diabetes. Rev Diabetic Stud.

[52] Niklasson B, Schønecker B, Bildsøe M, Rutledge E, Bekris L, Lernmark Ake (2003). Development of Type 1 Diabetes in Wild Bank Voles Associated With Islet Autoantibodies and the Novel Ljungan Virus. Experimental Diab. Res.

[53] Blixt M, Niklasson B, Sandler S (2007). Characterization of beta-cell function of pancreatic islets isolated from bank voles developing glucose intolerance/diabetes:an animal model showing features of both type 1 and type 2 diabetes mellitus, and a possible role of the Ljungan virus. Gen Comp Endocrinol.

[54] Niklasson B, Samsioe A, Blixt M, Lagerquist E, Lernmark Å, Klitz W (2006). Prenatal viral exposure followed by adult stress produces glucose intolerance in a mouse model. Diabetologia.

[55] Rook GA, Stanford JL (1998). Give us this day our daily germs. Immunol Today.

[56] Wen L, Ley R E, Volchkov P Y, Bluestone J.A (2008). Gordon JI, Chervonsky AV. Innate immunity and intestinal microbiota in the development of type diabetes. Nature.

[57] Duffy DL (2007). Genetic determinants of diabetes are similarly associated with other immune-mediated diseases. Curr Opin Allergy Clin Immunol.

[58] Huppmann M, Baumgarten A, Ziegler AG, Bonifacio E (2005). Neonatal Bacille Calmette-Guerin vaccination and type 1 diabetes. Diabetes Care.

[59] Heijbel H, Chen RT, Dahlquist G (1997). Cumulative incidence of childhood-onset IDDM is unaffected by pertussis immunization. Diabetes Care.

[60] Hyoty H, Hiltunen M, Reunanen A, Leinikki T, Vesikari T T, Akerblom Hk (1993). Decline of mumps antibodies in type 1 (insulin-dependent) diabetic children and a plateau in the rising incidence of type 1diabetes after introduction of the mumps-measles-rubella vaccine in Finland. Childhood Diabetes in Finland Study Group. Diabetologia.

[61] Risch N (1987). Assessing the role of HLA-linked and unlinked determinants of disease. Am J Hum Genet.

[62] Das A K, Sanjeevi CB, Shtauvere-Brameus A (2002). BCG vaccination and GAD65 and IA-2 autoantibodies in autoimmune diabetes in southern India. Ann NY Acad Sci.

[63] Baekkeskov S, Landin M, Kristensen JK, Srikanta S, Bruining GJ, Lindgren F (1987). Antibodies to a 64,000 Mr human islet cell antigen precede the clinical onset of insulin-dependent diabetes. J Clin Invest.

[64] Cai T, Xie J, She JX, Notkins AL (2001). Analysis of the coding and promoter regions of the autoantigen IA-2 in subjects with and without autoantibodies to IA-2. Diabetes.

[65] Bersani G, Zanco P, Padovan D, Betterle C (1981). Lymphocyte Subpopulations in insulin-dependent diabetics with and without serum islet cell autoantibodies. Diabetologia.

[66] Aanstoot HJ, Kang SM, Kim J, Lindsay LA, Roll U, Baekkeskov S (1996). Identification and characterization of glima 38,a glycosylated islet cell membrane antigen, which together with GAD65 and IA2 marks the early phases of autoimmune response in type 1diabetes. J Clin Invest.

[67] Gilliam LK, Lernmark A, Henry HL, Norman AW (2003). Diabetes Type 1 (Insulin-Dependent Diabetes Mellitus). Encyclopedia of Hormones. Seattle:Science Direct.

[68] Kaufman DL, Erlander MG, Clare-Salzler M, Atkinson MA, Maclaren NK, Tobin AJ (1992). Autoimmunity to two forms of glutamate decarboxylase in insulin-dependent diabetes mellitus. J Clin Invest.

[69] Kimpimaki T, Kulmala P, Savola K, Vahasalo P, Reijonen H, Knip M (2000). Disease-associated autoantibodies as surrogate markers of Type 1 diabetes in young children at increased genetic risk. Childhood Diabetes in Finland Study Group. J Clin Endocrinol Metab.

[70] Slover RH, Eisenbarth GS (1997). Prevention of type I diabetes and recurrent β cell destruction of transplanted islets. Endocr Rev.

[71] Bingley PJ, Bonifacio E, Gale EA (1993). Can we really predict IDDM?. Diabetes.

[72] Tandon N, Shtauvere-Brameus A, Hagopian WA, Sanjeevi CB (2002). Prevalence of ICA-12 and other autoantibodies in North Indian patients with early-onset diabetes. Ann. N.Y. Acad. Sci.

[73] Das AK, Shtauvere-Brameus A, Sanjeevi CB (2002). GAD65 and ICA antibodies in undernourished and normally nourished South Indian patients with diabetes. Ann. N.Y. Acad. Sci.

[74] Datta M, Shtauvere-Brameus A, Gupta V, Mani M K, Sanjeevi CB (2002). Autoimmune diabetes in 26 villages outside Madras. Ann. N.Y. Acad. Sci.

[75] Dabadghao P, Bhatia E, Bhatia V, Jayaraj K, Colmanb PG (1996). Islet-cell antibodies in malnutrition-related diabetes mellitus from North India. Diabetes Research and Clinical Practice.

[76] Christie MR, Tun RY, Lo SS, Cassidy D, Brown TJ, Leslie RD (1992). Antibodies to GAD and tryptic fragments of islet 64K antigen as distinct markers for development of IDDM:studies with identical twins. Diabetes.

[77] Schott M, Schatz D, Atkinson M, Krischer J, Mehta H, Maclaren N (1994). GAD65 autoantibodies increase the predictability but not the sensitivity of islet cell and insulin autoantibodies for developing insulin dependent diabetes mellitus. J Autoimmunity.

[78] Kelly MA, Rayner ML, Mijovic CH, Barnett AH (2003). Molecular aspects of type 1diabetes. Mol Pathol.

[79] Cudworth AG, Woodrow JC (1975). Evidence for HLA-linked genes in “juvenile” diabetes mellitus. Br Med J.

[80] Redondo MJ, Fain PR, Eisenbarth GS (2001). Genetics of type IA diabetes. Recent Prog Horm Res.

[81] Kim SJ, Jeong DG, Jeong SK, Yoon TS, Ryu SE (2007). Crystal structure of the major diabetes autoantigen insulinoma-associated protein 2 reveals distinctive immune epitopes. Diabetes.

[82] Solow H, Hidalgo R, Singal DP (1979). Juvenile-onset diabetes HLA-A, -B, -C and -DR alloantigens. Diabetes.

[83] Nerup J, Andersen OO, Bendixen G, Gunnarsson R, Kromann H, Poulsen JE (1974). Cell-mediated immunity in diabetes mellitus. Proc R Soc Med.

[84] Weitkamp LR (1981). HLA and disease:predictions for HLA haplotype sharing in families. Am J Hum Genet.

[85] Wolf E, Spencer KM, Cudworth AG (1983). The genetic susceptibility to type 1 (insulin-dependent) diabetes:analysis of HLA-DR association. Diabetologia.

[86] Todd JA, Bell JI, Mc Devitt HO (1987). HLA-DQ beta gene contributes to susceptibility and resistance to insulin-dependent diabetes mellitus. Nature.

[87] Corper AL, Stratmann T, Apostolopoulos V, Scott CA, Gracia KC, Teyton L (2000). A structural framework for deciphering the link between I-Ag7 and autoimmune diabetes. Science.

[88] Florez JC, Hirschhorn J, Altshuler D (2003). The inherited basis of diabetes mellitus:implications for the genetic analysis of complex traits. Annu Rev Genomics Hum Genet.

[89] Zamani M, Pociot F, Spaepen M, Raeymaekers P, Nerup J, Cassiman JJ (1996). Linkage and association of the HLA gene complex with IDDM in 81 Danish families:strong linkage between DR beta 1Lys71+ and IDDM. JMed Genet.

[90] Kockum I, Sanjeevi CB, Eastman S, Landin-Olsson M, Dahlquist G, Lernmark A (1995). Population analysis of protection by HLA-DR and DQ genes from insulin-dependent diabetes mellitus in Swedish children with insulin-dependent diabetes and controls. Eur J Immunogenet.

[91] Park Y (2006). Why is type 1 diabetes uncommon in Asia?. Ann N Y Acad Sci.

[92] Weintrob N, Sprecher E, Israel S, Pinhas-Hamiel O, Arbel A, Vardi P (2001). Type 1 diabetes environmental factors and correspondence analysis of HLA class II genes in the Yemenite Jewish community in Israel. Diabetes Care.

[93] Hermann R, Turpeinen H, Laine AP, Veijola R, Knip M, Ilonen J (2003). HLA DR-DQ-encoded genetic determinants of childhood-onset type 1 diabetes in Finland:An analysis of 622nuclear families. Tissue Antigens.

[94] Park YS, Wang CY, Ko KW, Yang SW, Park M, She JX (1998). Combinations of HLA DR and DQ molecules determine the susceptibility to insulin-dependent diabetes mellitus in Koreans. Hum Immunol.

[95] Murao S, Makino H, Kaino Y, Konoue E, Ohashi J, Osawa H (2004). Differences in the contribution of HLA-DR and –DQ haplotypes to susceptibility to adult and childhood-onset type 1 diabetes in Japanese patients. Diabetes.

[96] Rajalingam R, Ge P, Reed EF (2004). A sequencing-based typing method for HLA-DQA1 alleles. Human Immunol.

[97] Rani R, Sood A, Goswami R (2004). Molecular basis of predisposition to develop type 1diabetes mellitus in North Indians. Tissue Antigens.

[98] Shtauvere A, Kanungo A, Samal KC, Tripathi BB, Sanjeevi CB (1999). Association of HLA class II alleles with different subgroups of diabetes mellitus in Eastern India identify different associations with IDDM and malnutrition-related diabetes. Tissue Antigens.

[99] Witt CS, Kaur G, Cheong K, Kanga U, Christiansen F, Mehra NK (2002). Common HLA-B8-DR3 haplotype in Northern India is different from that found in Europe. Tissue Antigens.

[100] Torn C, Gupta M, Zake NL, Sanjeevi CB, Landin-Olsson M (2003). Heterozygosity for MICA5.0/MICA5.1 and HLA-DR3-DQ2/DR4-DQ8 are independent genetic risk factors for latent autoimmune diabetes mellitus in adults. Human Immunology.

[101] Rani R, Sood A, Lazaro AM, Stastny P (1999). Associations of MHC class II alleles with insulin-dependent diabetes mellitus (IDDM) in patients from North India. Hum Immunol.

[102] Mehra NK, Jaini R, Rajalingam R, Balamurugan A, Kaur G (2001). Molecular diversity in Asian Indians:predominance of A*0211. Tissue Antigens.

[103] Mehra NK, Kaur G, Kanga U, Tandon N (2002). Immunogenetics of autoimmune diseases in Asian Indians. Ann. N.Y. Acad. Sci.

[104] Mehra NK, Kumar N, Kaur G, Kanga U, Tandon N (2007). Biomarkers of susceptibility to type 1 diabetes with special reference to the Indian population. Indian J Med Res.

[105] Nepom GT, Erlich H (1991). MHC class-II molecules and autoimmunity. Ann Rev Immunol.

[106] Kawabata Y, Ikegami H, Kawaguchi Y, Fujisawa T, Hotta M, Ogihara T (2000). Age-related association of MHC class I chain-related gene a (MICA) with type 1 (Insulin-Dependent) diabetes mellitus. Human Immunology.

[107] Torn C, Gupta M, Sanjeevi CB, Aberg A, Frid A, Landin-Olsson M (2004). Different HLA-DR-DQ and MHC Class I Chain-Related Gene A (MICA) Genotypes in Autoimmune and Nonautoimmune Gestational Diabetes in a Swedish Population. Human Immunology.

[108] Gambelunghe G, Ghaderi M, Tortoioli C, Sanjeevi CB, Falorni A (2001). Umbria Type 1 diabetes Registry. Two distinct MICA gene markers discriminate major autoimmune diabetes types. J Clin Endocrinol Metab.

[109] Park Y, Lee H, Sanjeevi CB, Eisenbarth GS (2001). MICA Polymorphism Is Associated With type 1 Diabetes in the Korean Population. Diabetes Care.

[110] Alizadeh BZ, Eerligh P, van der Slik AR, Shastry A, Sanjeevi CB, Koeleman BP (2007). MICA marks additional risk factors for Type 1 diabetes on extended HLA haplotypes:an association and meta-analysis. Mol Immunol.

[111] Field SF, Nejentsev S, Walker NM, Howson JM, Godfrey LM, Todd JA (2008). Sequencing-based genotyping and association analysis of the MICA and MICB genes in type 1 diabetes. Diabetes.

[112] Byrne MM, Sturis J, O'Meara NM, Polonsky KS, Le-Roith Derek, Simeon I Taylor, Jerrold M Olefsky (1996). Insulin secretion in humans:Physiologic regulation and alteration in disease states - In Diabetes mellitus.

[113] Desai M, Zeggini E, Horton VA, Owen KR, Hattersley AT, Clark A (2006). The variable number of tandem repeats upstream of the insulin gene is a susceptibility locus for latent autoimmune diabetes in adults. Diabetes.

[114] Doria A, Lee J, Warram JH, Krolewski AS (1996). Diabetes susceptibility at IDDM2 cannot be positively mapped to the VNTR locus of the insulin gene. Diabetologia.

[115] Bennett ST, Lucassen AM, Gough SC, Powell EE, Undlien DE, Pociot F (1995). Susceptibility to human type 1diabetes at IDDM2 is determined by tandem repeat variation at the insulin gene minisatellite locus. Nat Genet.

[116] Bennett ST, Todd JA (1996). Human Type 1 diabetes and the insulin gene:principles of mapping polygenes. Annu Rev Genet.

[117] Bennett ST, Wilson AJ, Esposito L, Bouzekri N, Undlien DE, Todd JA (1997). Insulin VNTR allele-specific effect in type 1diabetes depends on identity of untransmitted paternal allele. The IMDIAB Group. Nat Genet.

[118] Vafiadis P, Ounissi-Benkalha H, Palumbo M, Grabs R, Goodyer CG, Polychronakos C (2001). Class III alleles of the variable number of tandem repeat insulin polymorphism associated with silencing of thymic insulin predispose to type 1 diabetes. J Clin Endocrinol Metab.

[119] Cox NJ, Wapelhorst B, Morrison VA, Johnson L, Todd JA, Concannon P (2001). Seven regions of the genome show evidence of linkage to type 1diabetes in a consensus analysis of 767 multiplex families. Am J Hum Genet.

[120] Kavvoura FK, Ioannidis JP (2005). CTLA-4 Gene Polymorphisms and Susceptibility to type 1 Diabetes Mellitus:A HuGE Review and Meta-Analysis. Am J Epidemiol.

[121] Zalloua PA, Abchee A, Shbaklo H, Zreik TG, Terwedow H, Azar ST (2004). Patients with early onset of type 1 diabetes have significantly higher GG genotype at position 49 of the CTLA4 gene. Hum Immun.

[122] Abe T, Takino H, Yamasaki H, Awata T, Yamaguchi Y, Eguchi K (1999). CTLA4 gene polymorphism correlates with the mode of onset and presence of ICA512 Ab in Japanese type 1 diabetes. Diabetes Res Clin Pract.

[123] Sedimbi SK, Kanungo A, Shastry A, Park Y, Sanjeevi CB (2007). No association of SUMO4 M55V with autoimmune diabetes in Asian-Indian patients Studies. Int J Immunogenet.

[124] Todd JA, Walker NM, Cooper JD, Dunger DB, Clayton DG, Wellcome Trust Case Control Consortium (2007). Robust associations of four new chromosome regions from genome-wide analyses of type 1 diabetes. Nat Genet.

[125] Sedimbi SK, Shastry A, Park Y, Rumba I, Sanjeevi CB (2006). Association of SUMO4 M55V polymorphism with autoimmune diabetes in Latvian patients. Ann N Y Acad Sci.

[126] Noso S, Ikegami H, Asano K, Hiromine Y, Awata T, Ogihara T (2006). Association of SUMO4, as a candidate gene for IDDM5, with susceptibility to type 1 diabetes in Asian populations. Ann N Y Acad Sci.

[127] Sedimbi SK, Sanjeevi CB, Lernmark A, Dahlquist G, Aman J, Swedish Childhood Diabetes Study Group, Diabetes Incidence in Sweden Study Group (2007). SUMO4 M55V polymorphism affects susceptibility to type I diabetes in HLA DR3- and DR4-positive Swedish patients. Genes Immun.

[128] The Wellcome Trust Case Control Consortium (2007). Genome-wide association study of 14,000 cases of seven common diseases and 3,000 shared controls. Nature.

[129] Vaessen N, Heutink P, Houwing-Duistermaat JJ, Snijders PJ, Rademaker T, Oostra BA (2002). A genome-wide search for linkage-disequilibrium with type 1 diabetes in a recent genetically isolated population from the Netherlands. Diabetes.

[130] Nerup J, Pociot F, European Consortium for IDDM studies (2001). A genome wide scan for type 1 diabetes susceptibility in Scandinavian families:identification of new loci with evidences of interactions. Am J Hum Genet.

[131] Concannon P, Erlich HA, Pociot F, Todd JA, Rich SS, Type 1 Diabetes Genetics Consortium (2005). Type 1 diabetes:evidence for susceptibility loci from four genome-wide linkage scans in 1,435 multiplex families. Diabetes.

[132] Smyth DJ, Cooper JD, Bailey R, Savage DA, Walker NM, Todd JA A genome-wide association study of nonsynonymous SNPs identifies a type 1 diabetes locus in the interferon-induced helicase (IFIH1) region. Nature genetics.

[133] Liu S, Wang H, Jin Y, Eisenbarth G, Rewers M, She JX (2009). IFIH1 polymorphisms are significantly associated with type 1 diabetes and IFIH1 gene expression in peripheral blood mononuclear cells. Hum Mol Genet.

[134] American Diabetes Association (2003). Prevention of type 1 diabetes mellitus. Diabetes care.

[135] Devendra D, Liu E, Eisenbarth GS (2004). Type 1 diabetes:recent developments. BMJ.

[136] Tian C, Bagley J, Cretin N, Seth N, Wucherpfennig KW, Iacomini J (2004). Prevention of type 1 diabetes by gene therapy, J. Clin. Invest.

[137] Diabetes prevention trial –type 1 diabetes study group (2002). Effects of insulin in relatives of patients with type 1 diabetes mellitus. NEJM.

[138] Skyler JS (2007). Prediction and prevention of type 1 diabetes:progress, problems, and prospects. Clin Pharmacol Ther.

[139] Palmer JP, Fleming GA, Greenbaum CJ, Polonsky KS, Skyler JS, Steffes MW (2004). C-peptide is the appropriate outcome measure for type 1 diabetes clinical trials to preserve beta-cell function:report of an ADA workshop. Diabetes.

[140] Sosenko JM, Cowie C, Greenbaum CJ, Cuthbertson D, Lachin JM, Skyler JS, Diabetes Prevention Trial-Type 1 Study Group (2008). A risk score for type diabetes derived from autoantibody-positive participants in the diabetes prevention trial-type 1. Diabetes Care.

[141] Winter WE, Schatz D (2003). Prevention strategies for type 1 diabetes mellitus:current status and future directions. Bio Drugs.

[142] Keymeulen B (2006). New therapies aimed at the preservation or restoration of beta cell function in type 1 diabetes. Acta Clin Belg.

[143] Keymeulen B (2008). Therapies aimed at preservation or restoration of beta cell function in type 1 diabetes. Verh K Acad Geneeskd Belg.

[144] Petrovsky N, Silva D, Schatz DA (2003). Vaccine therapies for the prevention of type 1 diabetes mellitus. Paediatr Drugs.

[145] Staeva-Vieira T, Peakman M, von Herrath M (2007). Translational mini-review series on type 1 diabetes:Immune-based therapeutic approaches for type 1 diabetes. Clin Exp Immunol.

[146] Kishiyama CM, Chase HP, Barker JM (2006). Prevention strategies for type 1 diabetes. Rev Endocr Metab Disord.

